# Sex dictates IL-17A regulation: Inflammatory determinants in males versus a potassium-linked metabolic axis in females

**DOI:** 10.1371/journal.pone.0341044

**Published:** 2026-05-18

**Authors:** David Chisompola, Emmanuel Luwaya, Martin Chakulya, Joseph M. Chalwe, Joreen P. Povia, Annet Kirabo, Sepiso K. Masenga

**Affiliations:** 1 Department of Cardiovascular Science and Metabolic Diseases, Livingstone Center for Prevention and Translational Science, Livingstone, Zambia; 2 HAND Research Group, School of Medicine and Health Sciences, Mulungushi University, Livingstone, Zambia; 3 Nuclear Medicine Research Infrastructure (NuMeRI), Capital Park, Pretoria, South Africa; 4 Department of Nuclear Medicine, University of Pretoria and Steve Biko Academic Hospital, Pretoria, South Africa; 5 Department of Health Economics, Livingstone Center for Prevention and Translational Science, Livingstone, Zambia; 6 Department of Medicine, Vanderbilt University Medical Center, Nashville, Tennessee, United States of America; 7 Vanderbilt Center for Immunobiology, Vanderbilt Institute for Infection, Immunology and Inflammation, Vanderbilt University Medical Center, Nashville, Tennessee, United States of America; 8 Department of Molecular Physiology and Biophysics, Vanderbilt University Medical Center, Nashville, Tennessee, United States of America; 9 Vanderbilt Institute for Global Health, Vanderbilt University Medical Center, Nashville, Tennessee, United States of America; Yakin Dogu Universitesi, TÜRKIYE

## Abstract

**Background:**

Interleukin-17A (IL-17A) is a key Th17 cytokine involved in mucosal defense and chronic inflammation and serves as an important biomarker and therapeutic target in autoimmune, cardiovascular, and infectious diseases. Emerging evidence indicates that immune regulation may differ by sex, which may influence disease susceptibility, biomarker interpretation, and treatment response. However, few studies have examined sex-specific determinants of IL-17A. This study aimed to identify the sociodemographic, clinical, and inflammatory correlates of circulating IL-17A, with a specific focus on elucidating sex-specific determinants.

**Methods:**

We conducted a cross-sectional study in a cohort of adults attending routine clinic from Livingstone University Teaching Hospital in Zambia. Plasma levels of IL-17A and a panel of inflammatory cytokines were measured. Sociodemographic, metabolic and clinical data, including HIV status and ART regimen, were collected. Sex-stratified multiple linear regression models were used to identify independent correlates of IL-17A levels with statistical significance p < 0.05.

**Results:**

A total of 225 participants were recruited, comprising 71 males and 154 females, with median ages 50 (41, 59) vs. 48 (40, 58) years, respectively. Distinct sex-specific factors associated with IL-17A were identified. In males, IL-17A levels were significantly associated with a recognized inflammatory cytokine network, including positive correlations with IL-6 (Beta: 43.96, 95% CI: 30.58–57.34, p < 0.001) and IL-1β (Beta: 0.02, 95% CI: 0.02–0.03, p < 0.001), and a negative association with IFN-γ (Beta: −1.21, 95% CI: −1.65 – −0.77, p < 0.001). However, living with HIV was not a predictor of IL-17A (Beta: −1248.07, 95% CI: −2752.35–256.21, p = 0.101). Among females, none of the cytokines or HIV status predicted IL-17A; instead, plasma potassium (Beta: 9.81, 95% CI: 5.31–14.30, p < 0.001) was the only significant determinant of IL-17A.

**Conclusion:**

The determinants of IL-17A are fundamentally different between males and females. These findings underscore the importance of considering biological sex as a key variable in immunology research. They suggest that IL-17A may require sex-specific interpretation as a biomarker of inflammation, particularly in HIV-associated and cardiometabolic conditions, and highlight the potential for sex-tailored therapeutic strategies targeting IL-17A-related pathways. Further studies are needed to validate these findings and explore their mechanistic and clinical implications.

## Introduction

Interleukin-17A (IL-17A) is a key cytokine orchestrating host defense, inflammation, and autoimmune pathology, primarily produced by T-helper 17 (Th17) cells [1]. Its production is classically regulated by a network of inflammatory cytokines, including IL-6 and IL-1β, which promote Th17 differentiation, and IFN-γ, which can antagonize this pathway [2,3]. While IL-17A is a well-established mediator in chronic inflammatory and autoimmune conditions, its regulation in the context of hypertension, human immunodeficiency virus (HIV) infection and the potential influence of biological sex remains incompletely understood.

HIV infection and antiretroviral therapy (ART) are associated with complex immune dysregulation and persistent inflammation, which contribute to an increased risk of non-AIDS comorbidities [4]. The Th17/IL-17A axis is of particular interest, as Th17 cells are crucial for mucosal immunity and are preferentially depleted in HIV infection, with implications for immune recovery and comorbid disease risk [5]. Furthermore, there is compelling evidence that highlights profound sex differences in immune responses, disease susceptibility, and inflammatory outcomes [6,7]. These differences are driven by a combination of genetic, hormonal, and environmental factors, suggesting that the pathways governing a key cytokine like IL-17A may also be sex-specific.

IL-17A is a well-established inflammatory mediator that promotes vascular dysfunction and contributes significantly to the pathogenesis of hypertension [8,9]. Although the global incidence of hypertension is rising, driving increased research into its underlying immunological mechanisms [8], a notable gender disparity persists. Men are affected at a higher rate (15.51%) than women (11.22%) [10]. Despite the recognized role of immune mediators like IL-17A, the specific immunological factors underlying this sex disparity remain unclear.

In addition to classical cytokine-mediated pathways, emerging evidence suggests that electrolyte balance may influence immune function. Potassium flux, in particular, plays a key role in regulating NLRP3 inflammasome activation, which governs IL-1β production, a critical upstream driver of Th17 differentiation and IL-17A secretion [11,12]. While this pathway has been primarily described in experimental models, it highlights a potential link between potassium homeostasis and IL-17A regulation. Notably, sex differences in immune responses are influenced by hormonal and physiological factors, including estrogen-mediated modulation of immune signaling pathways [13,14]. In addition, sex hormones contribute to differences in renal electrolyte handling, including potassium regulation, which may further shape immune responses in a sex-specific manner [15,16]. However, the relationship between potassium and IL-17A in human populations, and whether this association differs by sex, remains poorly understood.

Furthermore, most immunological studies either do not stratify by sex or are underpowered to detect sex-specific mechanisms, potentially obscuring critical biological insights. This gap is significant, as a failure to account for sex as a biological variable may lead to incomplete models of disease pathogenesis and the development of non-optimized therapeutics [17].

Although IL-17A regulation has been extensively studied, most prior investigations have not examined sex-specific determinants. Many studies have either combined male and female participants without stratified analyses or have been limited by relatively small sample sizes, reducing power to detect sex-dependent associations [18,19]. In addition, existing research has largely focused on canonical cytokine pathways, with limited exploration of non-traditional factors such as metabolic or electrolyte influences. These gaps may have obscured important sex-specific regulatory mechanisms of IL-17A.

Therefore, we hypothesized that the sociodemographic, metabolic, and inflammatory determinants of circulating IL-17A would differ fundamentally between males and females. In a well-characterized cohort of Zambian adults with and without HIV, we aimed to identify these sex-specific regulatory pathways by integrating data on cytokines, metabolic profiles, electrolytes, and clinical status. Elucidating these pathways is essential for reinterpreting IL-17A as a context- and sex-dependent biomarker and for advancing precision medicine in chronic inflammatory and infectious diseases.

## Materials and methods

### Study design and population

A cross-sectional study was conducted involving 225 adults who attended routine medical checkup at a tertiary hospital in Livingstone, Zambia.

### Eligibility criteria

Participants were eligible if they were aged 18 years or older and provided written informed consent. To minimize confounding effects on inflammatory markers, we excluded individuals with diabetes, pregnancy, severe renal or hepatic disease and active opportunistic infections.

### Sample size determination

The sample size of 225 participants was considered adequate for multivariable regression analyses based on established recommendations of at least 10–15 observations per predictor variable to ensure model stability and avoid overfitting [20,21]. In addition, this sample size provided sufficient power to detect moderate effect sizes in sex-stratified analyses, although smaller associations may not have been detectable.

### Data collection

Data collection commenced on the 25^th^ of August 2023–30^th^ April 2024.

### Sociodemographic and clinical data

Sociodemographic and clinical data were systematically collected and managed using the Research Electronic Data Capture (REDCap) platform. Trained research personnel gathered information on age, sex, marital status, employment status, and medical history (including HIV, diabetes, hypertension, and cardiovascular conditions) through standardized structured interviews and medical record reviews. HIV status and ART regimen details were confirmed from clinical records.

### Biochemical measurements

Following an overnight fast of 8–12 hours, venous blood samples were obtained from each participant using sterile vacutainer tubes. Samples collected for cytokine assays were drawn into EDTA tubes, whereas serum separator tubes (SST) were used for biochemical analyses. All samples were transported to the laboratory on ice within 30 minutes of collection to minimize pre-analytical degradation. Plasma was prepared by centrifuging the EDTA tubes at 2,500 rpm for 10 minutes at 4°C, and the supernatant was carefully aliquoted into pre-labeled cryovials. Serum samples were allowed to clot for 20–30 minutes at room temperature before undergoing centrifugation under identical conditions. All aliquots were stored at −80°C until batch analysis, and multiple freeze–thaw cycles were avoided to preserve sample integrity.

Cytokine quantification for IL-17A, IL-1β, IL-5, IL-6, TNF-α, and IFN-γ was performed using commercially available enzyme-linked immunosorbent assay (ELISA) (Elabscience, Houston, USA) kits in accordance with the manufacturers’ protocols. The lower limits of detection (LOD) for the cytokines were as follows: IL-17A (7.81pg/mL), IL-6 (7.81 pg/mL), IL-1β (7.81 pg/mL), and TNF-α (7.81pg/mL), with inter- and intra-assay CVs of <8% and <10%. Prior to analysis, frozen plasma samples were thawed on ice and mixed gently to ensure homogeneity. Standards, quality controls, and appropriately diluted plasma samples were added to antibody-coated microplates and incubated to allow antigen–antibody binding. Absorbance was measured at 450 nm using calibrated microplate reader, and cytokine concentrations were calculated from four-parameter logistic standard curves. Low and high assay controls were included on each plate to ensure analytical precision and reproducibility.

### Statistical analysis

Data were exported from the REDCap platform into a structured format and underwent initial cleaning in Microsoft Excel to ensure data integrity. All statistical analyses were performed using StatCrunch. Descriptive statistics were presented as medians with interquartile ranges (IQR) for continuous variables and frequencies with percentages for categorical variables. The relationship between IL-17A and covariates was first assessed using simple linear regression, with results visualized in scatter plots. Multivariable regression models were constructed by including variables that demonstrated statistical significance (p < 0.05) in bivariate analyses. This approach was used to achieve model parsimony and reduce the risk of overfitting given the sample size. A p-value of < 0.05 was considered statistically significant.

Prior to regression analyses, key model assumptions were assessed. Normality of residuals was evaluated using visual inspection of Q–Q plots and histograms. Multicollinearity was assessed using variance inflation factors (VIF), with values >5 indicate moderate collinearity, and values >10 indicate high collinearity [22]. Homoscedasticity was assessed using the Breusch–Pagan/Cook–Weisberg test. Where heteroscedasticity was detected, regression models were re-estimated using robust standard errors to ensure valid statistical inference.

During model development, variables demonstrating high multicollinearity were evaluated, and IL-5 was excluded from the final multivariable model to improve model stability and interpretability.

Missing data were minimal across all variables. Analyses were conducted using available-case (pairwise) methods, whereby all available observations were included in each analysis.

Given the exploratory nature of the analyses and the use of multivariable regression models to assess independent associations, formal correction for multiple testing was not applied. Findings were interpreted in the context of biological plausibility and existing literature.

### Ethical considerations

Prior to commencing the study, ethical approval and administrative permissions were secured from the following bodies: the Mulungushi University School of Medicine and Health Sciences Ethics Committee (MUHSREC) on 09 July 2023 (Ref: SMHS-MU3-2023-005). All participants provided written informed consent after the study’s objectives, methodology, potential risks, and benefits were thoroughly explained to them in a comprehensible language. Participation was entirely voluntary, and strict measures were implemented to safeguard data confidentiality and participant privacy throughout the research. No participant identifiers were collected.

## Results

### Sociodemographic and clinical profiles

A total of 225 participants (71 males, 154 females) were included in this analysis, with median ages of 50 (IQR 41–59) and 48 (IQR 40–58) years, respectively. The cohorts differed significantly in several sociodemographic characteristics. A higher proportion of males were married (70.4% vs. 32.5%) and employed (42.3% vs. 30.5%). Clinical profiles revealed several significant disparities. Females had a higher median BMI (25.9 vs. 21.9 kg/m², p < 0.001). Heart failure was more frequently reported among males (7.2% vs. 2.2%, p = 0.046), while diabetes was more prevalent in males (6.2% vs. 2.2%, p = 0.027). Smoking was also more common in males (5.7% vs. 3.4%, p = 0.011). The prevalence of hypertension was similar between groups. Notable differences were also observed in laboratory parameters. Males demonstrated significantly lower median levels of triglycerides, LDL cholesterol, and VLDL, as well as a lower cholesterol-HDL ratio (all p < 0.001). Inflammatory markers also varied significantly: females had higher median levels of IFN-γ, IL-5, and IL-6 (p < 0.001 and p = 0.001, respectively), while males had higher median levels of TNF-α and IL-1 (both p < 0.001). All significant findings are detailed in Table 1.

**Table 1:** Sociodemographic, clinical, inflammatory and metabolic and kidney correlates of IL-17a.

Variable	Males = 71		Females = 154		P value
	Frequency (n)	Percent (%)	Frequency (n)	Percent (%)	
**Age (years), IQR**	50 (41, 59)		48 (40, 58)		**<0.001**
**Marital status**					
*Married*	50	70.4	50	32.5	0.980
*Not married*	21	29.6	104	67.5	
**Employment status**					
*Employed*	30	42.3	47	30.5	0.957
*Not employed*	41	57.7	107	69.5	
**HIV Status**					
*Positive*	41	57.7	104	67.1	1.000
*Negative*	30	42.3	51	32.9	
**ART Regimen**					
*NNRTI+NRTI*	0	0	4	4.6	1.000
*INSTI (TLD or TafED)*	39	100	81	93.1	
*PIs*	0	0	2	2.3	
**ART Adherence**					
*Yes*	10	55.6	28	77.8	1.000
*No*	8	44.4	8	22.2	
**Diabetes Status**					
*Diabetic*	4	6.2	3	2.2	**0.027**
*Nondiabetic*	61	93.8	132	97.8	
**Hypertension status**					
*Hypertensive*	23	32.4	43	27.7	0.967
*Non-hypertensive*	48	67.6	112	72.3	
**Antihypertensive drugs taking**					
*0 drug*	2	11.1	1	2.9	0.998
*1 drug*	11	61.1	17	48.6	
*2 drugs*	3	16.6	16	45.7	
*3 drugs*	2	11.1	0	0	
*4 drugs*	0	0	1	2.8	
**Hypertensive Crisis on or Off**					
*Yes*	2	2.9	5	3.4	0.529
*No*	68	97.1	141	96.6	
**Hypertensive Heart Disease**					
*Yes*	7	10.1	16	11.3	0.249
*No*	62	89.9	125	88.7	
**Heart Failure**					
*Yes*	5	7.2	3	2.2	**0.046**
*No*	64	92.8	136	97.8	
**Do you feel any Palpitations**					
*Yes*	16	23.9	51	36.4	1.000
*No*	51	76.1	89	63.6	
**Do you think you eat salt**					
*Yes*	22	33.3	30	21.3	0.854
*No*	44	66.7	111	78.7	
**History of TB**					
*Yes*	9	15.3	17	13.6	0.364
*No*	50	84.7	108	86.4	
**Smoking**					
*Yes*	4	5.7	5	3.4	**0.011**
*No*	66	94.3	143	96.6	
**BMI (kg/m2), IQR**	21.9 (19.8, 26.4)		25.9 (21.8, 29.9)		**<0.001**
**Triglycerides, IQR**	0.7 (0.5, 1.1)		0.8 (0.5, 1.2)		**<0.001**
**LDL Cholesterol, IQR**	2.6 (2.2, 3.4)		3.1 (2.5, 3.8)		**<0.001**
**VLDL, IQR**	0.3 (0.2, 0.5)		0.3 (0.2, 0.5)		**<0.001**
**Cholesterol HDL Ratio, IQR**	3.4 (2.8, 4.3)		3.7 (3.0, 4.6)		**<0.001**
**D dimer, IQR**	3479.5 (1983.9, 11417.0)		3860 (2068.9, 13024.1)		**<0.001**
**IFN-γ (ng), IQR**	1309 (28.2, 2213.5)		1516.7 (25.1, 2346.8)		**<0.001**
**IL-6 (pg/mL), IQR**	10.6 (0.8, 108.8)		86.1 (0.8, 108.7)		**0.001**
**TNF-a (pg/mL), IQR**	1569.7 (34.2, 3587.8)		557.4 (27.2, 3324.3)		**<0.001**
**IL-1 (pg/mL, IQR)**	6.2 (3.1, 183.8)		3.6 (2.5, 225)		**<0.001**
**IL-5 (pg/mL), IQR**	285.3 (13.1, 1114.1)		327.7 (11.8, 1426.3)		**<0.001**
**Fasting Glucose (mmol/L), IQR**	4.8 (4.2, 5.3)		4.8 (4.4, 5.5)		**<0.001**
**Plasma Potassium (mmol/L), IQR**	4.1 (3.9, 4.4)		4.0 (3.8, 4.3)		**<0.001**

**Abbreviations**: IQR, Interquartile Range; ART, Antiretroviral Therapy; NNRTI, Non-Nucleoside Reverse Transcriptase Inhibitor; NRTI, Nucleoside Reverse Transcriptase Inhibitor; INSTI, Integrase Strand Transfer Inhibitor; TLD, Tenofovir/Lamivudine/Dolutegravir; TafED, Tenofovir alafenamide/Emtricitabine/Dolutegravir; PI, Protease Inhibitor; TB, Tuberculosis; BMI, Body Mass Index; LDL, Low-Density Lipoprotein; VLDL, Very Low-Density Lipoprotein; HDL, High-Density Lipoprotein; IFN, Interferon; IL, Interleukin; TNF-α, Tumor Necrosis Factor-alpha. Data are presented as median (IQR), or n (%). The number of observations may vary across variables due to minimal missing data. Analyses were performed using available-case (pairwise) methods.

### Simple linear regression in Males

**Fig 1:**
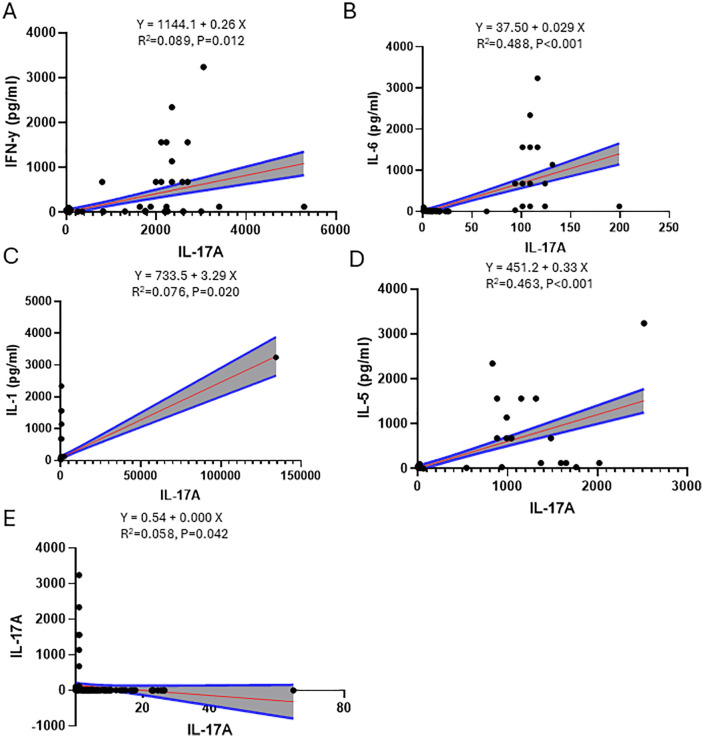
Males’ simple linear regression.

Simple linear regression analyses in male participants identified several factors significantly associated with IL-17A levels. The strongest positive associations were with IL-6 (R² = 0.488, p < 0.001; Fig 1B) and IL-5 (R² = 0.463, p < 0.001; Fig 1D). More modest positive associations were found for IFN-γ (R² = 0.089, p = 0.012; Fig 1A) and IL-1 (R² = 0.076, p = 0.020; Fig 1C). HIV status demonstrated a weak but statistically significant relationship (R² = 0.058, p = 0.042; Fig 1E).

### Simple linear regression in Females

**Fig 2:**
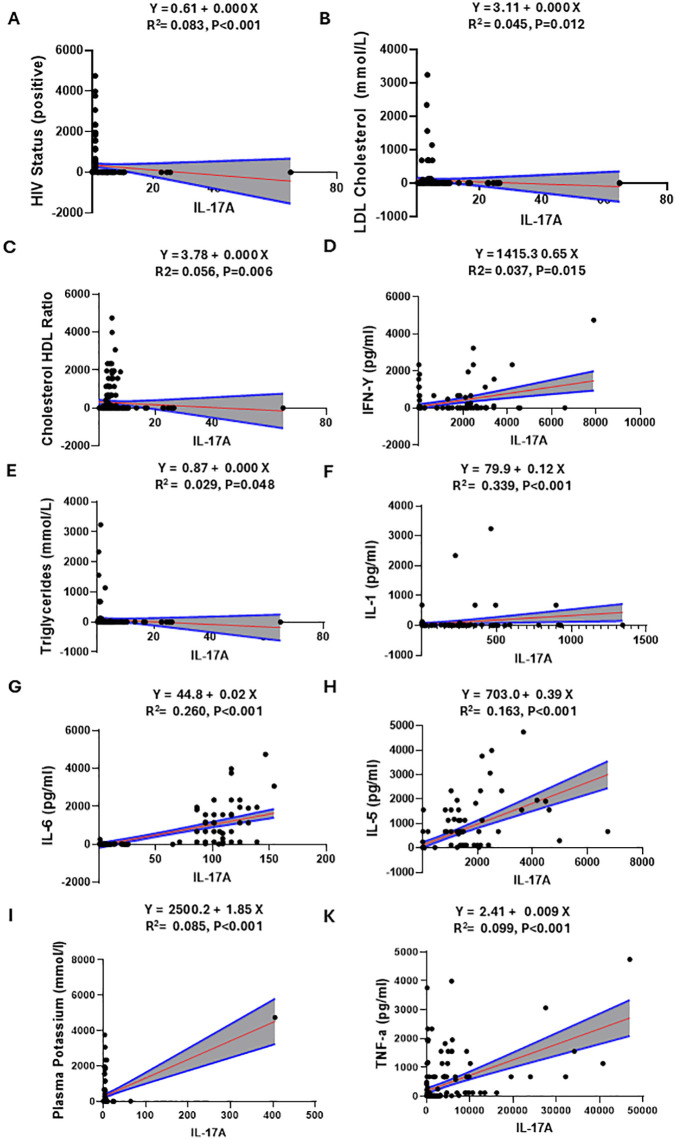
Females’ Simple linear regression.

Among female participants, IL-17A levels showed significant associations in simple linear regression models. The strongest predictors were IL-1 (R² = 0.339, p < 0.001; Fig 2F) and IL-6 (R² = 0.260, p < 0.001; Fig 2G). Weaker but significant positive associations were observed for plasma potassium (R² = 0.099, p < 0.001; Fig 2J), HIV status (R² = 0.083, p < 0.001; Fig 2A), TNF-α (R² = 0.085, p < 0.001; Fig 2I), cholesterol: HDL ratio (R² = 0.056, p = 0.006; Fig 2C), LDL cholesterol (R² = 0.045, p = 0.012; Fig 2B), IFN-γ (R² = 0.037, p = 0.015; Fig 2D), and triglycerides (R² = 0.029, p = 0.048; Fig 2E).

### Association of inflammatory markers with IL-17A in males

In the multiple linear regression model for males, which included all variables significant in simple linear regression analyses, IL-6 (β = 43.96, 95% CI: 37.06 to 50.86, p < 0.001) and IL-1β (β = 0.02, 95% CI: 0.01 to 0.03, p < 0.001) demonstrated significant positive associations with IL-17A levels (Table 2). Conversely, IFN-γ (β = −1.18, 95% CI: −1.57 to −0.79, p < 0.001) showed significant negative associations. However, HIV-positive status (β = −1248.07, 95% CI: −2752.35 to 256, p = 0.101) was not statistically significant (Table 2). Initial models included IL-5; however, due to evidence of multicollinearity (VIF > 10), this variable was excluded from the final model, resulting in improved model stability (S1 Table).

**Table 2:** Males’ multiple linear regression.

Parameter	Beta	95% L. Limit	95% U. Limit	P-Value
HIV status (positive)	−1248.07	−2752.35	256.21	0.101
IL-6 (pg/mL)	43.96	30.58	57.34	**<0.001**
IFN-γ (pg/mL)	−1.18	−1.88	−0.48	**0.001**
IL-1 (pg/mL)	0.02	0.02	0.03	**<0.001**

The Breusch–Pagan test indicated evidence of heteroscedasticity (χ^2^ = 10.3, p = 0.001). Regression models were therefore fitted using robust standard errors. The use of robust estimation did not materially alter the direction or significance of the associations observed.

### Association of inflammatory markers and metabolic factors with IL-17A in females

In the multiple linear regression model for females, which included all variables significant in simple linear regression analyses. The multiple linear regression model adjusted for inflammatory markers and metabolic profiles which revealed that none of the cytokines or HIV status were significant predictors of IL-17A levels. Instead, plasma potassium was the only significant independent determinant, showing a positive association (β = 9.81, 95% CI: 5.31 to 14.30, p < 0.001) (Table 3). IL-5 was excluded from the final multivariable model due to multicollinearity identified during model diagnostics (S2 Table).

**Table 3:** Females’ multiple linear regression.

Parameter	beta	95% L. Limit	95% U. Limit	P-value
HIV status (positive)	222.8	−147.63	593.19	0.235
Triglycerides (mmol/L)	118.8	−212.22	449.83	0.477
LDL Cholesterol (mmol/L)	194.2	−101.15	489.49	0.195
Cholesterol HDL Ratio	−3.74	−209.69	202.20	0.971
IFN-γ (pg/mL)	0.01	−0.06	0.08	0.771
IL-1 (pg/mL)	0.85	−0.06	0.08	0.771
IL-6 (pg/mL)	5.88	−0.65	2.35	0.264
TNF-a (pg/mL)	−0.02	−0.08	0.03	0.366
Plasma potassium (mmol/L)	9.81	5.31	14.30	**<0.001**

The Breusch–Pagan test indicated strong evidence of heteroscedasticity (χ^2^ = 47.43, p < 0.001). Therefore, robust standard errors were applied in this regression model.

## Discussion

This study reveals distinct sex-based differences in sociodemographic, clinical, and inflammatory characteristics among the study cohort, as well as differing predictors of IL-17A levels between males and females. The study findings suggest a more tightly regulated, cytokine-driven inflammatory network in males, where HIV status and key inflammatory mediators such as IL-6, IFN-γ, and IL-1, were significantly associated with IL-17A levels. However, IL-17A regulation in females appeared to be less dependent on inflammatory pathways and more closely linked to metabolic factors, particularly plasma potassium levels. These results underscore fundamental sex-specific mechanisms in immune regulation and point to the potential influence of non-inflammatory physiological systems on IL-17A in females. These differing findings align with known biology by which IL-17A production in males is closely integrated into the recognized Th17-mediated inflammation, where IL-6 and IL-1 promote Th17 differentiation, and IFN-γ antagonizes Th17 pathways [23–26].

In females, however, none of the classical inflammatory cytokines predicted IL-17A levels. While, plasma potassium emerged as the only significant correlate, other variables such as lipid profiles, cytokines, and HIV status were not. This novel association may reflect known interactions between electrolytes and immune signaling, where potassium efflux modulates inflammasome activation and downstream cytokine responses [27,28]. Given that potassium homeostasis can be influenced by hormonal fluctuations, this association may represent a hormonally mediated immunometabolism mechanism. Moreover, the absence of traditional inflammatory drivers further suggests that IL-17A regulation in females may follow non-canonical metabolic or hormonal pathways, potentially influenced by estrogen-driven immunomodulation, which is known to alter Th17 responses [29].

The observed association between plasma potassium and IL-17A in females warrants careful interpretation. Although not traditionally considered a primary regulator of adaptive immune responses, potassium plays a critical role in innate immune signaling. In particular, potassium efflux is a well-established trigger of NLRP3 inflammasome activation, which promotes the release of IL-1β, a key upstream cytokine driving Th17 differentiation and IL-17A production [28,30]. In addition, ion channel activity has been increasingly recognized as an important regulator of immune cell activation, proliferation, and cytokine secretion [31]. Potassium gradients across the cell membrane can influence T cell receptor signaling and downstream transcriptional responses. Furthermore, emerging evidence suggests that electrolyte balance may interact with cellular metabolic pathways, including mitochondrial function and redox signaling, which are known to shape T cell differentiation and effector function.

HIV status showed a negative association with IL-17A in males but not in females, indicating possible sex-related differences in mucosal immune recovery or Th17 cell depletion under ART [32]. The universal use of INSTI-based regimens among males may further contribute to these differences, as some INSTIs have been linked to shifts in cytokine signaling [33]. In females, the lack of association between HIV and IL-17A suggests that HIV-related immune dysregulation is either attenuated or masked by stronger metabolic or hormonal influences [34]. This sex-specific pattern highlights the need to evaluate ART response beyond conventional viral suppression metrics.

Mechanistically, the male pattern is consistent with the canonical IL-6/IL-1β–Th17 axis, in which IL-6 drives STAT3-dependent Th17 differentiation and IL-1β enhances IL-17A-producing responses [35]. In contrast, the female pattern may reflect upstream regulation at the level of ion flux and immunometabolism, as potassium efflux is a recognized trigger of inflammasome activation and may influence downstream pathways linked to IL-17A biology [28]. These data therefore suggest that IL-17A in males may function mainly as a marker of active inflammatory cytokine signaling, whereas in females it may be linked more closely to electrolyte-sensitive cellular regulation.

The observed sex-specific patterns may also have important implications for the interpretation of IL-17A as a biomarker. In males, where IL-17A was closely linked to IL-6 and IL-1β, it likely reflects an active pro-inflammatory cytokine network and may therefore serve as a marker of systemic inflammatory burden. This is consistent with existing literature in which IL-6, IL-1β, and IL-17A are co-regulated and associated with inflammatory and cardiovascular risk pathways [36,37]. In contrast, in females, the association of IL-17A with potassium rather than classical cytokines suggests that IL-17A may reflect upstream regulatory processes rather than overt inflammation. As such, IL-17A levels in females may not directly correspond to circulating inflammatory cytokine activity, but instead capture more subtle immunometabolic or electrolyte-linked regulation.

These differences may have clinical relevance in conditions characterized by chronic immune activation, such as HIV infection and cardiovascular disease. In people living with HIV, persistent immune activation involving IL-6, IL-17A, and related pathways has been linked to increased cardiovascular risk and endothelial dysfunction [38]. Similarly, IL-17A has been implicated in vascular inflammation and atherosclerosis through its interactions with IL-6 and other downstream mediators. Our findings suggest that the interpretation of IL-17A as a biomarker in these conditions may need to consider sex-specific regulatory contexts. In males, IL-17A may more directly reflect inflammatory disease activity, whereas in females, its interpretation may require consideration of non-traditional regulatory factors such as electrolyte balance. These observations highlight the importance of sex-stratified approaches in biomarker research and may have implications for risk stratification and therapeutic targeting.

Although females had higher BMI and LDL cholesterol, these did not predict IL-17A, highlighting that general metabolic inflammation may not directly shape Th17 activity in women within this cohort. Males, who experienced stronger cytokine-mediated IL-17A regulation, showed lower BMI and higher smoking rates, factors known to alter systemic inflammation and oxidative stress, potentially amplifying cytokine interactions [39].

The inclusion of BMI and lipid parameters in our analysis also provides an opportunity to interpret IL-17A within the broader context of immunometabolism. Increasing evidence indicates that IL-17A is closely linked to metabolic dysregulation, particularly in obesity and metabolic syndrome. Adipose tissue expansion is associated with increased Th17 cell activity and IL-17A production, contributing to a state of chronic low-grade inflammation [40]. In addition, IL-17A has been shown to influence lipid metabolism and insulin sensitivity, potentially exacerbating metabolic dysfunction [41]. Dyslipidemia, including elevated triglycerides and altered cholesterol profiles, has similarly been associated with inflammatory pathways involving IL-17A and related cytokines.

These relationships suggest that IL-17A may act at the interface between immune and metabolic systems, linking inflammation with cardiometabolic risk. In this context, the associations observed in our study between IL-17A and metabolic parameters such as BMI and lipid profiles may reflect underlying immunometabolic processes rather than isolated inflammatory signals [42]. This is particularly relevant given the established contribution of metabolic dysfunction to cardiovascular disease, where IL-17A and Th17 responses have been implicated in vascular inflammation and atherosclerosis. Taken together, these findings support the interpretation of IL-17A as a biomarker that integrates both inflammatory and metabolic dimensions of disease.

These findings have important clinical and research implications. First, sex must be treated as a critical biological variable in IL-17A-related research, given the observed divergence in its correlates and regulatory pathways between males and females. Second, in clinical practice, the interpretation of IL-17A as a biomarker, particularly within HIV-affected populations, may require sex-specific reference ranges to improve diagnostic and prognostic accuracy. Third, the unexpected association between plasma potassium and IL-17A in females highlights a novel, non-inflammatory regulatory axis that warrants further mechanistic investigation into electrolyte–immune crosstalk. Finally, the male-specific interaction between HIV status and IL-17A suggests possible sex differences in gut barrier integrity, mucosal Th17 homeostasis, or antiretroviral immune recovery, which could inform more tailored management of HIV-related inflammation and its comorbidities. Collectively, these insights advocate for a precision medicine approach that incorporates sex-specific pathophysiology in both immunological research and patient care.

This study has several limitations that should be considered when interpreting the findings. First, the cross-sectional design limits the ability to infer causality between IL-17A levels and the observed inflammatory, metabolic, or clinical correlates. Longitudinal studies would be required to determine temporal relationships and mechanistic pathways. Second, although the sample size was adequate for exploratory modeling, the unequal distribution of males and females may have reduced statistical power to detect sex-specific associations, particularly among males. Third, cytokine measurements were based on single time-point plasma samples, which may not fully capture biological fluctuations or circadian variation in immune markers. Given the number of variables examined, the possibility of Type I error cannot be excluded, and findings should be interpreted with caution. However, the consistency of key associations and their biological plausibility support the robustness of the main findings.

Additionally, potential confounders such as diet, hormonal status such as menstrual cycle phase or menopausal status, micronutrient deficiencies, and co-infections were not evaluated but may influence IL-17A responses differently between sexes. ART regimens, although documented, were heterogeneous in females, and ART duration and adherence levels could not be fully quantified, potentially affecting immune recovery patterns. The study was conducted in a single urban setting, which may limit generalizability to rural or more diverse populations. Finally, although multiple cytokines and metabolic markers were included in the models, unmeasured pathways, such as gut microbiota, mucosal immunity, and genetic polymorphisms, may contribute to IL-17A regulation and could explain some of the sex-specific differences observed. Future studies designed to address these limitations will be crucial to validate and extend these provocative findings.

## Conclusion

This study highlights marked sex differences in IL-17A regulation. In males, IL-17A is driven primarily by classical inflammatory cytokines within a coherent Th17 axis. In females, IL-17A appears decoupled from cytokine networks and instead is influenced by electrolyte balance. These sex-specific differences underscore the need for tailored immunological assessments and may inform more personalized approaches to managing chronic inflammation in HIV-affected populations

## Supporting information

S1 File STROBE. (DOCX)

S1 Data Data. (XLSX)

S1 Table Variance inflation factors (VIF) in males. (DOCX)

S2 Table Variance inflation factors (VIF) in females. (DOCX)
