# Supplementary material for: Sex dictates IL-17A regulation: Inflammatory determinants in males versus a potassium-linked metabolic axis in females
Source: PLoS One. 2026 May 18;21(5):e0341044. doi: 10.1371/journal.pone.0341044 (PMC13183186; doi:10.1371/journal.pone.0341044)
Supplement: S1 Table — (DOCX) [file pone.0341044.s003.docx]

**S1 Table. Variance inflation factors (VIF) in Males**

| **Variables** | **VIF** | **1/VIF** |
| --- | --- | --- |
| HIV status (Positive) | 4.77 | 0.209 |
| IFN-y (pg/mL) | 6.31 | 0.158 |
| IL6 (pg/mL) | 5.23 | 0.191 |
| IL-1 (pg/mL) | 1.08 | 0.927 |
| **Mean VIF** | **4.35** |  |
| **Abbreviations**: VIF, variance inflation factor. VIF was used to assess multicollinearity among predictor variables in the regression models. VIF values >5 indicate moderate collinearity, and values >10 indicate high collinearity that may affect the stability and interpretability of regression coefficients. IL-5 was excluded from the final regression model due to high multicollinearity. | | |
