# Supplementary material for: Sex dictates IL-17A regulation: Inflammatory determinants in males versus a potassium-linked metabolic axis in females
Source: PLoS One. 2026 May 18;21(5):e0341044. doi: 10.1371/journal.pone.0341044 (PMC13183186; doi:10.1371/journal.pone.0341044)
Supplement: S2 Table — (DOCX) [file pone.0341044.s004.docx]

**S2 Table. Variance inflation factors (VIF) in Females**

| **Variable** | **VIF** | **1/VIF** |
| --- | --- | --- |
| IL-6 (pg/mL) | 4.95 | 0.202 |
| HIV Status (positive) | 4.5 | 0.222 |
| cholesterol | 3.29 | 0.304 |
| IL-1 (pg/mL) | 2.4 | 0.417 |
| LDL Cholesterol (mmol/l) | 2.18 | 0.459 |
| Triglycerides (mmol/l) | 1.91 | 0.523 |
| TNF-a (pg/mL) | 1.72 | 0.583 |
| Plasma Potassium (mmol/l) | 1.55 | 0.646 |
| IFN-gamma (pg/mL) | 1.12 | 0.894 |
| **Mean VIF** | 2.62 |  |
| **Abbreviations**: VIF, variance inflation factor. VIF was used to assess multicollinearity among predictor variables in the regression models. VIF values >5 indicate moderate collinearity, and values >10 indicate high collinearity that may affect the stability and interpretability of regression coefficients. IL-5 was excluded from the final regression model due to high multicollinearity. | | |
